# Variations in soil microbial community structure and extracellular enzymatic activities along a forest–wetland ecotone in high‐latitude permafrost regions

**DOI:** 10.1002/ece3.10205

**Published:** 2023-06-15

**Authors:** Lingyu Fu, Ruifeng Xie, Dalong Ma, Man Zhang, Lin Liu

**Affiliations:** ^1^ College of Geographical Sciences Harbin Normal University Harbin China

**Keywords:** ecotone, extracellular enzymatic activities, microbial community structure, permafrost, wetland

## Abstract

Permafrost degradation by global warming is expected to alter the hydrological processes, which results in changes in vegetation species composition and gives rise to community succession. Ecotones are sensitive transition areas between ecosystem boundaries, attract particular interest due to their ecological importance and prompt responses to the environmental variables. However, the characteristics of soil microbial communities and extracellular enzymes along the forest–wetland ecotone in high‐latitude permafrost region remain poorly understood. In this study, we evaluated the variations of soil bacterial and fungal community structures and soil extracellular enzymatic activities of 0–10 cm and 10–20 cm soil layers in five different wetland types along environmental gradients, including *Larix gmelinii* swamp (LY), *Betula platyphylla* swamp (BH), *Alnus sibirica* var. *hirsute* swamp (MCY), thicket swamp (GC), and tussock swamp (CC). The relative abundances of some dominant bacterial (Actinobacteria and Verrucomicrobia) and fungal (Ascomycota and Basidiomycota) phyla differed significantly among different wetlands, while bacterial and fungal alpha diversity was not strongly affected by soil depth. PCoA results showed that vegetation type, rather than soil depth explained more variation of soil microbial community structure. β‐glucosidase and β‐N‐acetylglucosaminidase activities were significantly lower in GC and CC than in LY, BH, and MCY, while acid phosphatase activity was significantly higher in BH and GC than LY and CC. Altogether, the data suggest that soil moisture content (SMC) was the most important environmental factor contributing to the bacterial and fungal communities, while extracellular enzymatic activities were closely related to soil total organic carbon (TOC), nitrate nitrogen (${\mathrm{NO}}_{3}^{-}\mathrm{–N}\;$) and total phosphorus (TP).

## Graphical abstract

We evaluated the variations of soil bacterial and fungal community structures and soil extracellular enzymatic activities of 0–10 cm and 10–20 cm soil layers in five different wetland types along environmental gradients. This study may enhance our comprehensive understanding of the complex microbial processes in high‐latitude permafrost region wetland ecosystems under climate change scenarios.

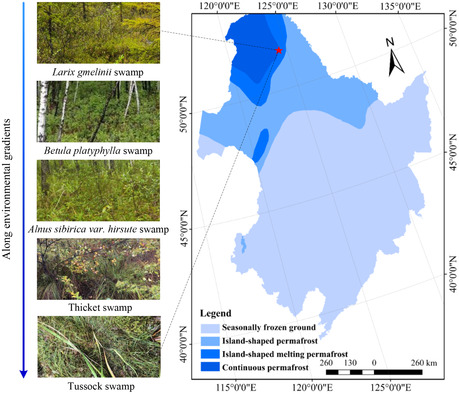

## 1 INTRODUCTION

Due to climate warming and anthropogenic disturbances, wetlands have degraded at an astonishing speed on a global scale. This trend not only severely influences the balance of local plant communities, soil, hydrology, and overall wetland ecosystems but also threatens regional ecological security (Lu et al., 2021). High northern latitudes distribute more than 50% of the global wetlands, where permafrost is also prevalent that has a strong effect on wetland hydrology (Avis et al., 2011). Permafrost has the ability to prevent vertical drainage and retain soil moisture, and its integrity contributes to the sustainability of wetlands in cold regions (Lawrence et al., 2015). Owing to rising temperatures in high‐latitude regions, permafrost has partly degraded, resulting in the deepening of the soil active layer and in a decrease of wetland water table, which in turn affects key wetland ecosystem processes such as soil thermal regimes, carbon and nitrogen dynamics, transportation of nutrients, and transformation of contaminants (Smith et al., 2022; Xue et al., 2021).

Soil microorganisms are an important component of wetland ecosystems, and these have a vital role in decomposition of organic matter, humus formation, material circulation, and energy flow, as well as controlling the differentiation and succession of wetland types (Yarwood, 2018). Bacteria and fungi together account for more than 90% of soil microbial biomass, whereas their abilities to degrade complex and available forms of organic matter differ (López‐Mondéjar et al., 2020). Synergism of these different microbial members promotes plant diversity and growth by adding different limiting nutrients to the soil, thereby driving crucial wetland ecosystem functions. Soil extracellular enzymes, produced by plant roots and microorganisms, function as biological catalysts; these are not only crucial for microbial nutrient acquisition and soil elemental balances, but are also considered as indispensable indicators of soil nutrient resource limitations and microbial energy metabolism (Chen et al., 2021). Ecotones, transition zones occurring where the environmental limits of species and communities overlap, have been suggested as valuable areas for monitoring early shifts in community structure due to human activities and climate changes (Vowles et al., 2018). Forest–wetland ecotones are areas with sharp environmental gradients, providing an important filter on the distribution and composition of microbial and plant communities (Meng et al., 2021). Therefore, a deep investigation of the functions of bacterial and fungal communities in the evolution of forest–wetland ecotone is of significance for the conservation and restoration of cold region wetlands and is essential to effectively addressing climate change.

The Nanweng River Nature Reserve at the southern boundary of the Eurasian permafrost is the sole nature reserve in China that protects cold temperate forest‐wetland ecosystems. It is reported that this permafrost region has significantly degraded in the recent past, so that the southern boundary has retreated 20–30 km northwards since the 1960s (Jin et al., 2000). The degradation of permafrost alters the hydrology, topography and vegetation succession of wetlands at high latitudes, and further affects the stability of the carbon pool and the process of greenhouse gas emissions in wetlands (Kreplin et al., 2021; Mu et al., 2020). Microorganisms are critical players to predict the potential feedback mechanisms between high‐latitude permafrost wetlands and climate systems. Deepening active layer under the warming climate leads to the flow of shallow surface water to deeper layers, and a possible shift in wetland vegetation structure from hygrophilous community to xeromorphic or shrub community, which is expected to release labile nutrients, increase microbial activities, and enhance decomposition rates (Xue et al., 2021). However, knowledge of how the continuous transitions between vegetation types affect soil microbial community diversity, structure, and metabolic potential in the forest–wetland ecotone is still limited. In this study, five typical natural wetlands (*Larix gmelinii* swamp, *Betula platyphylla* swamp, *Alnus sibirica* var. *hirsute* swamp, thicket swamp and tussock swamp) in the Nanweng River Nature Reserve were selected, which were distributed successively along the forest–wetland ecotone in high‐latitude permafrost region. Soil environmental factors, microbial (bacterial and fungal) communities and extracellular enzymatic activities in both the upper (0–10 cm) and lower (10–20 cm) soil layers were measured to better explore wetland ecosystem stability and evolutionary processes in high‐latitude permafrost region. We hypothesized that (1) microbial community structure and extracellular enzymatic activities would follow the patterns of change associated with wetland vegetation type, and (2) microbial communities show a weaker response to soil depth, but a stronger relationship with vegetation type. This study may enhance our comprehensive understanding of the complex microbial processes in high‐latitude permafrost region wetland ecosystems under climate change scenarios.

## 2 MATERIALS AND METHODS

### 2.1 Study area and soil sampling

**FIGURE 1:**
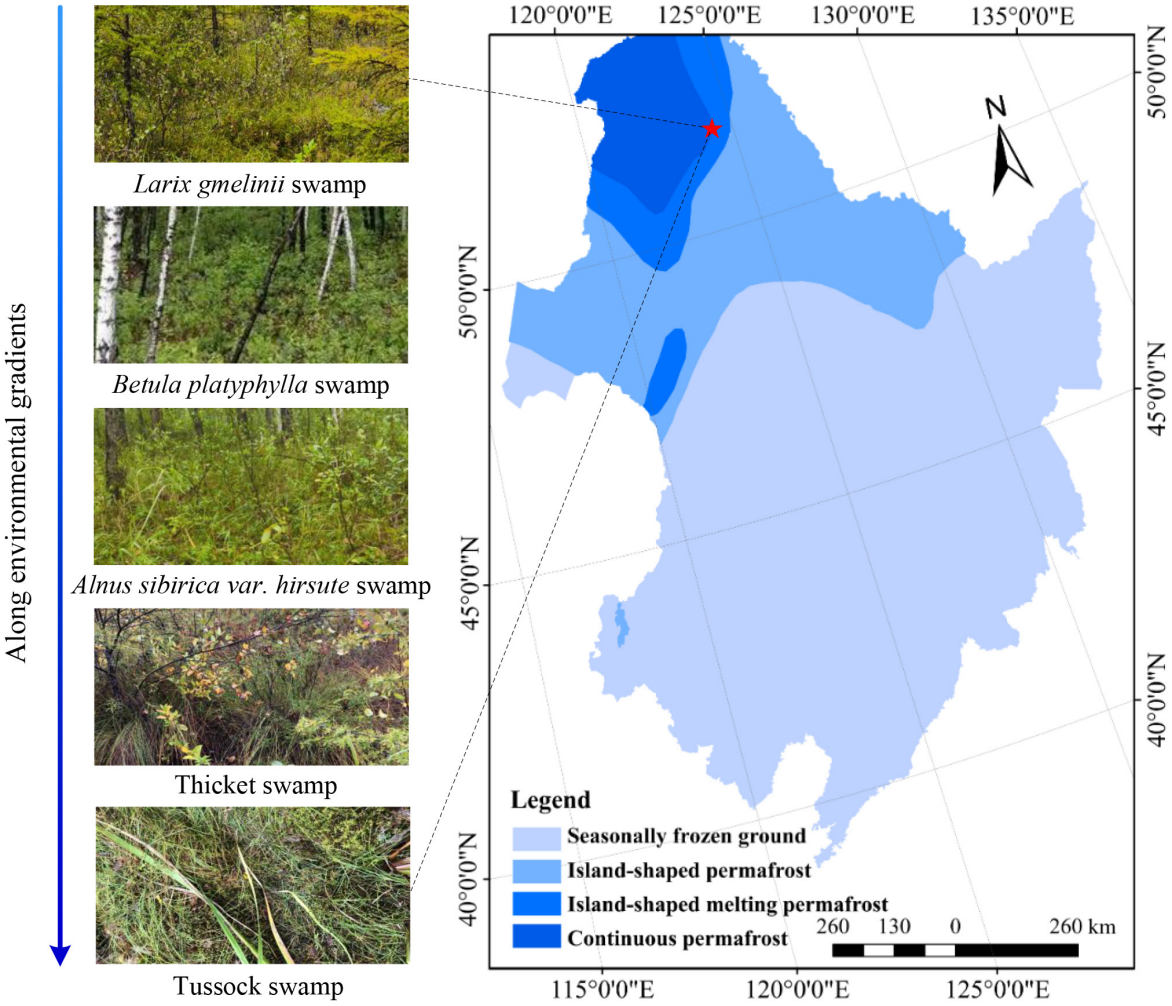
Map of the sampling sites.

The study area is located at the Nanweng River Nature Reserve (51°05′07″–51°39′24″N, 125°07′55″–125°50′05″E) in the discontinuous permafrost zone of the Great Xing'an mountains, China. The local continental monsoon climate in this cold temperate zone has an average annual temperature of −3°C and an extreme minimum temperature of −48°C. The frost‐free period is 90–100 d, and the annual precipitation is 500 mm, most of which occurs during July–August. Soils are mainly brown coniferous forest soils, and meadow soil, bog soil, and peat soil are also distributed in this area (Yu et al., 2013). Permafrost and periglacial phenomena in the study area form a characteristic cold and wet ecosystem combining forests and marshes and preserved various plant species native to the Great Xing'an mountains terrestrial, aquatic and bog biotopes. The wetlands typically overlie permafrost, including forest wetlands, shrub wetlands, grass wetlands, marsh wetlands, and island forest wetlands. On the basis of extensive field investigations and monitoring, we selected five representative fixed experimental plots in the forest–wetland ecotone along environmental gradients (mainly water table, active layer depth, and vegetation type), including *Larix gmelinii* swamp (LY), *Betula platyphylla* swamp (BH), *Alnus sibirica* var. *hirsute* swamp (MCY), thicket swamp (GC), and tussock swamp (CC) (Figure 1). The mean water table depths were −43.6 cm, −35.9 cm, −27.1 cm, −18.5 cm, and −13.4 cm, respectively. The active layer thicknesses ranged from 50 cm to 190 cm. The dominant tree species are mainly including *Larix gmelinii*, *Betula platyphylla* and *Alnus sibirica* var. *hirsuta*, and the shrub layer is dominated by *Betula ovalifolia*, *Vaccinium uliginosum*, and *Ledum palustre*, while *Eriophorum vaginatum*, *Carex appendiculata*, and *Deyeuxia angustifolia* are largely distributed in the herb layer. For each of these plots, three 10 m × 10 m quadrats were randomly established, and five sampling points were defined in each quadrat according to the “S” route. On August 2021, soil samples were collected at 0–10 cm and at 10–20 cm depths using a soil auger (5 cm diameter). After removing coarse material such as gravel and plant roots, all soil samples from the same plot and soil layer were thoroughly mixed in sterilized ziplock bags to give one subsample, named “MCY10” for MCY soil at 0–10 cm depth, “GC20” for GC soil at 10–20 cm depth, etc. All subsamples were kept in an ice‐cooled box and immediately transported to the laboratory, where they were divided into three equal parts for further analysis. The first part was stored at −80°C for high‐throughput sequencing, the second part was stored at 4°C for the measurement of soil extracellular enzymatic activities, and the third part was passed through a 0.5 mm sieve and used to determine the soil physicochemical properties.

### 2.2 Soil physicochemical analysis

To determine the soil moisture content (SMC), a fraction of the fresh, sieved soil was weighed, then dried at 105°C for 24 h and weighed again; the difference in mass was used to calculate SMC. Soil pH was measured by a pH meter at a water‐to‐soil mass ratio of 2.5:1. Total organic carbon (TOC) was quantified using a Multi N/C 3100 analyzer (Analytik Jena AG, Germany) by the combustion method. Total nitrogen (TN) was measured using the automatic Kjeldahl apparatus (Kjeltec 8400, Danmark). Total phosphorus (TP) was determined by molybdenum blue colorimetric method following H_2_SO_4_–HClO_4_ digestion. Soil nitrate nitrogen (${\mathrm{NO}}_{3}^{-}$–N) and ammonium nitrogen (${\mathrm{NH}}_{4}^{+}$–N) were determined using a continuous flow analyzer (Skalar SAN^++^, Netherlands).

### 2.3 DNA extraction and PCR amplification

Total DNA was extracted from the soil samples using the PowerSoil DNA Isolation Kit (MOBIO, USA). The V3–V4 region of the bacterial 16S rRNA gene were amplified by PCR with the primers 338F (5′‐ACTCCTACGGGAGGCAGCAG‐3′) and 806R (5′‐GGACTACHVGGGTWTCTAAT‐3′). The ITS2 region of the fungal gene was amplified by PCR with the primers FITS7 (5′‐GTGARTCATCGAATCTTTG‐3′) and ITS4 (5′‐TCCTCCGCTTATTGATATGC‐3′). All PCR amplifications were performed in triplicate and triplicate amplicons were pooled and then checked by 2% agarose gel electrophoresis. The purified amplicons were quantified using Qubit (Invitrogen, USA). The purified PCR products were checked for library quality using Agilent 2100 Bioanalyzer (Agilent Technologies, USA). Illumina Miseq high‐throughput sequencing was conducted at Lianchuan Biotechnology Co., Ltd (Hangzhou, China). After filtering low‐quality and ambiguous base sequences in raw paired‐end reads using Trimmomatic software and joining using FLASH software (Magoč & Salzberg, 2011), chimeras were removed using the UCHIME algorithm (Edgar et al., 2011). The resulting high‐quality sequences were used for subsequent analysis. Operational taxonomic units (OTUs) were clustered with a 97% similarity threshold using Vsearch (v.2.3.4). The RDP classification algorithm was used to classify the obtained 16S rRNA and ITS fragment sequences based on the SILVA (v.128) and UNITE (v.7.1) databases (Tremblay & Yergeau, 2019), respectively.

### 2.4 Soil extracellular enzyme assays

Soil microbial community function was quantified by analyzing the potential activities of five extracellular enzymes, which were determined within 1 week of soil storage at 4°C. The analyses included two C‐cycling enzymes (β‐glucosidase, BG and cellobiohydrolase, CB), two N‐cycling enzymes (β‐N‐acetylglucosaminidase, NAG and leucine aminopeptidase, LAP), and one P‐cycling enzyme (acid phosphatase, AP). These were assessed with fluorometric assays based on the protocol of Saiya‐Cork et al. (2002). Briefly, 1.0 g of soil (dry weight equivalent) was homogenized with 125 mL sodium acetate buffer (50 mm, pH 5.0), and 200 μL of the suspension was combined with 50 μL of fluorometric substrate in each well of 96‐well microplates. The LAP activity was assessed using substrate analogs attached to fluorescent molecules 7‐amino‐4‐methylcoumarin (AMC), and the activities of rest enzymes (BG, CB, NAG, and AP) were assessed by substrate analogs attached to 4‐methylumbelliferone (MUB). Each assay was paired with a 10 μM AMC or MUB standard. Both the assay and the standard were performed for eight analytical replicates to elucidate soil heterogeneity. Following incubation at 20°C for 4 h, fluorescence was determined at 365 nm excitation and 450 nm emission using a multifunctional microplate reader (SpectraMax M5, USA). Enzyme activity was expressed as nmol/g dry weight/h, and the enzyme C:N, C:P, and N:P ratios were calculated by (BG + CB):(NAG+LAP), (BG + CB):AP, and (NAG + LAP):AP, respectively.

### 2.5 Statistical analysis

Alpha diversity indices (Chao1, Shannon, and phylogenetic diversity) were calculated using QIIME (v.1.9.1). Principal coordinate analysis (PCoA) was conducted to analyze the differences in microbial communities by Canoco software (v.5.0) (Šmilauer & Lepš, 2014). Permutational multivariate analysis of variance (PERMANOVA) was performed to test the differences across different wetlands (*vegan* package in R) (Dixon, 2003). Venn diagrams were drawn using the *VennDiagram* package in R software (Chen & Boutros, 2011). One‐way analysis of variance (ANOVA) was performed to determine differences in soil physicochemical properties and extracellular enzymatic activities between the different wetlands in SPSS software (v.20.0) (Xiao et al., 2021). Two‐way analysis of variance (ANOVA) was conducted to determine the effects of wetland type, soil depth, and their interactions on microbial alpha diversity. Pearson's correlation coefficients were conducted to examine the correlations among soil physicochemical properties, microbial community diversity, and extracellular enzymatic activities using SPSS software (v.20.0). Redundancy analysis (RDA) was conducted to analyze the relationships between soil physicochemical properties and microbial communities using Canoco software (v.5.0).

## 3 RESULTS

### 3.1 Soil physicochemical properties

Soil moisture content (SMC) tended to increase in the sampled soils from *Larix gmelinii* swamp (LY) to tussock swamp (CC), with the lowest value (49.36) determined in LY at 10–20 cm depth (LY20) and the highest (112.65) in CC20 (Table 1). The pH of the soil ranged from 4.86 to 5.92 and differed significantly between 0–10 cm and 10–20 cm soil layers in the different wetlands. Soil total organic carbon (TOC) showed a trend of first decreasing, then increasing, and then decreasing. TOC was markedly higher in CC and thicket swamp (GC) than in *Alnus sibirica* var. *hirsute* swamp (MCY), *Betula platyphylla* swamp (BH) and LY. Soil ammonium nitrogen (${\mathrm{NH}}_{4}^{+}$–N) and nitrate nitrogen (${\mathrm{NO}}_{3}^{-}$–N) contents were significantly higher at 0–10 cm depth than in the 10–20 cm soil layer, except for ${\mathrm{NH}}_{4}^{+}$–N in BH. Soil total nitrogen (TN) showed a tendency to decrease, then increase and then decrease, and MCY, GC, and CC had significantly higher TN contents than BH. The contents of total phosphorus (TP) were significantly lower in BH and GC than in CC.

**TABLE 1:** Soil physicochemical properties in different wetlands.

Sample	SMC (%)	pH	TOC (g/kg)	${\mathrm{NH}}_{4}^{+}$–N (mg/kg)	${\mathrm{NO}}_{3}^{-}$–N (mg/kg)	TN (g/kg)	TP (g/kg)
LY10	57.09 ± 8.84^Ca^	5.63 ± 0.17^Aa^	169.36 ± 9.15^Ca^	8.57 ± 2.28^Ca^	19.36 ± 1.62^Da^	11.02 ± 1.18^Ba^	0.81 ± 0.05^Bb^
LY20	49.36 ± 6.10^Db^	5.31 ± 0.08^Bb^	132.52 ± 7.48^Cb^	6.03 ± 0.93^Db^	12.21 ± 2.40^Cb^	6.38 ± 0.89^Cb^	1.03 ± 0.08^Aa^
BH10	61.25 ± 7.62^Ca^	5.29 ± 0.05^Ba^	143.25 ± 13.22^Ca^	11.96 ± 1.33^Ca^	28.47 ± 0.88^Ca^	9.55 ± 0.66^Ca^	0.45 ± 0.11^Cb^
BH20	65.81 ± 5.91^Ca^	4.86 ± 0.07^Cb^	121.08 ± 14.62^Cb^	9.75 ± 1.07^Ca^	20.95 ± 2.05^Bb^	5.09 ± 0.70^Cb^	0.59 ± 0.06^Ca^
MCY10	70.23 ± 6.21^Bb^	5.05 ± 0.18^Cb^	220.57 ± 8.20^Ba^	13.68 ± 0.80^Ca^	36.82 ± 3.83^Ba^	15.36 ± 2.10^Aa^	0.95 ± 0.09^ABa^
MCY20	82.02 ± 4.60^Ba^	5.92 ± 0.14^Aa^	166.91 ± 6.54^Bb^	11.05 ± 1.52^Cb^	30.63 ± 0.73^Ab^	14.25 ± 0.64^Aa^	0.83 ± 0.10^Ba^
GC10	88.63 ± 8.73^Ab^	5.52 ± 0.22^Aa^	296.63 ± 12.91^Aa^	22.59 ± 3.61^Ba^	45.18 ± 4.27^Aa^	13.42 ± 1.53^ABa^	0.57 ± 0.14^Cb^
GC20	103.54 ± 10.75^Aa^	5.15 ± 0.13^Bb^	237.42 ± 10.47^Ab^	17.96 ± 0.62^Bb^	23.96 ± 1.96^Bb^	13.08 ± 2.13^Aa^	0.80 ± 0.05^Ba^
CC10	92.77 ± 9.14^Ab^	5.37 ± 0.07^Bb^	273.26 ± 24.79^Aa^	30.78 ± 2.77^Aa^	39.76 ± 0.78^Ba^	12.25 ± 0.44^Ba^	1.22 ± 0.11^Aa^
CC20	112.65 ± 13.71^Aa^	5.68 ± 0.09^Aa^	225.11 ± 16.77^Ab^	23.55 ± 1.93^Ab^	32.33 ± 3.18^Ab^	11.11 ± 1.30^Ba^	1.15 ± 0.06^Aa^

*Note*: Values are mean ± SD; different capital letters denote significant differences between five wetlands at the same depth (*p* < .05); different lowercase letters denote significant differences between two soil depths in the same wetland (*p* < .05).

### 3.2 Bacterial and fungal community diversity

**FIGURE 2:**
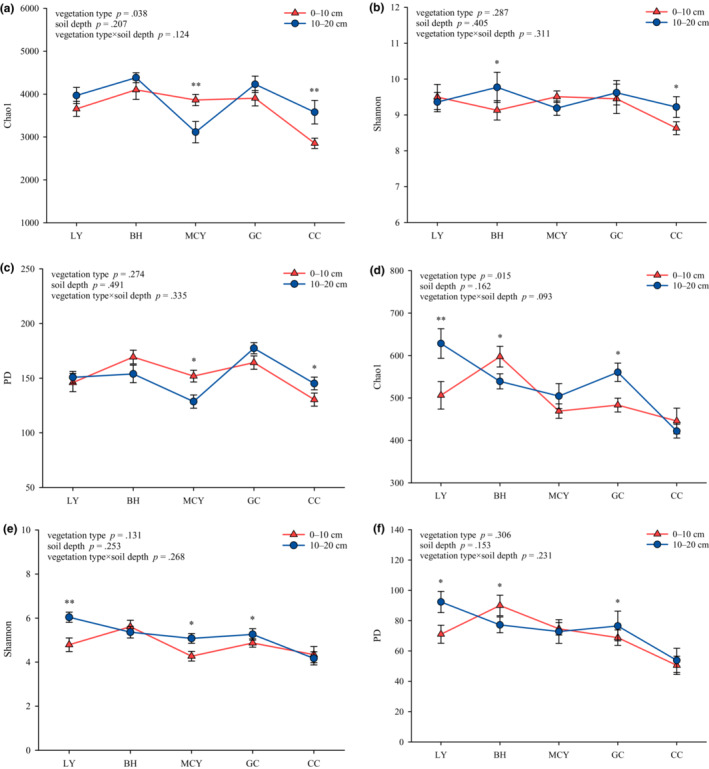
Soil microbial community alpha diversity. (a) Bacterial Chao1 estimator; (b) bacterial Shannon index; (c) bacterial PD index; (d) fungal Chao1 estimator; (e) fungal Shannon index; (f) fungal PD index. Various capital letters mean significantly different between five wetland types at the same depth (*p* < .05). *p < .05; **p < .01.

After removing all of low‐quality sequences, 1,547,736 bacterial and 1,836,093 fungal valid sequences were obtained. The species richness (Chao1 estimator) and Shannon diversity index of the bacterial community were both significantly higher in BH and GC than in CC, while the phylogenetic diversity (PD) index was markedly higher in GC than in LY, MCY and CC (*p* < .05) (Figure 2). Fungal Chao1 and PD indices were significantly lower in CC than others, and Shannon index was markedly lower in CC than in LY, BH, and GC (*p* < .05). Analysis of variance (ANOVA) identified significant differences in bacterial alpha diversity indices between CC10 and CC20. The bacterial Chao1 and PD indices were significantly higher in MCY10 than in MCY20 (*p* < .05). In addition, in LY and GC, fungal alpha diversity indices at 0–10 cm depth were significantly different from those at 10–20 cm depth (*p* < .05). Two‐way ANOVA results confirmed that bacterial and fungal Chao1 estimator were significantly affected by vegetation type (*p* < .05), while the Shannon and PD indices were not influenced by vegetation type, soil depth, and their interactions (*p* > .05).

**FIGURE 3:**
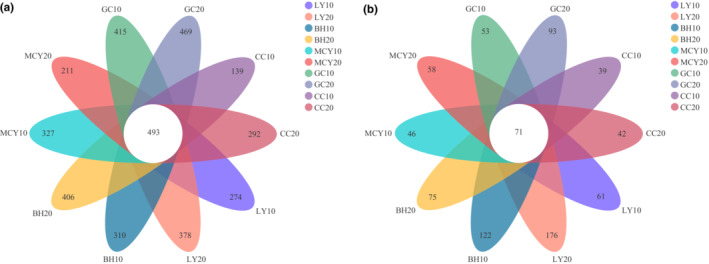
Venn diagrams of microbial community of soil samples in the five wetlands. (a) Bacterial communities; (b) fungal communities.

A total of 493 common bacterial OTUs were identified in all soil samples combined (Figure 3a). The highest number of unique bacterial OTUs was observed in GC20 (469), followed by GC10 (415) and the lowest was observed in CC10 (139). However, only a total of 71 fungal OTUs were shared among all different wetland soil samples (Figure 3b). The number of unique fungal OTUs in LY20 was the highest (176), followed by BH10 (122), and the lowest was detected in CC10 (39).

**FIGURE 4:**
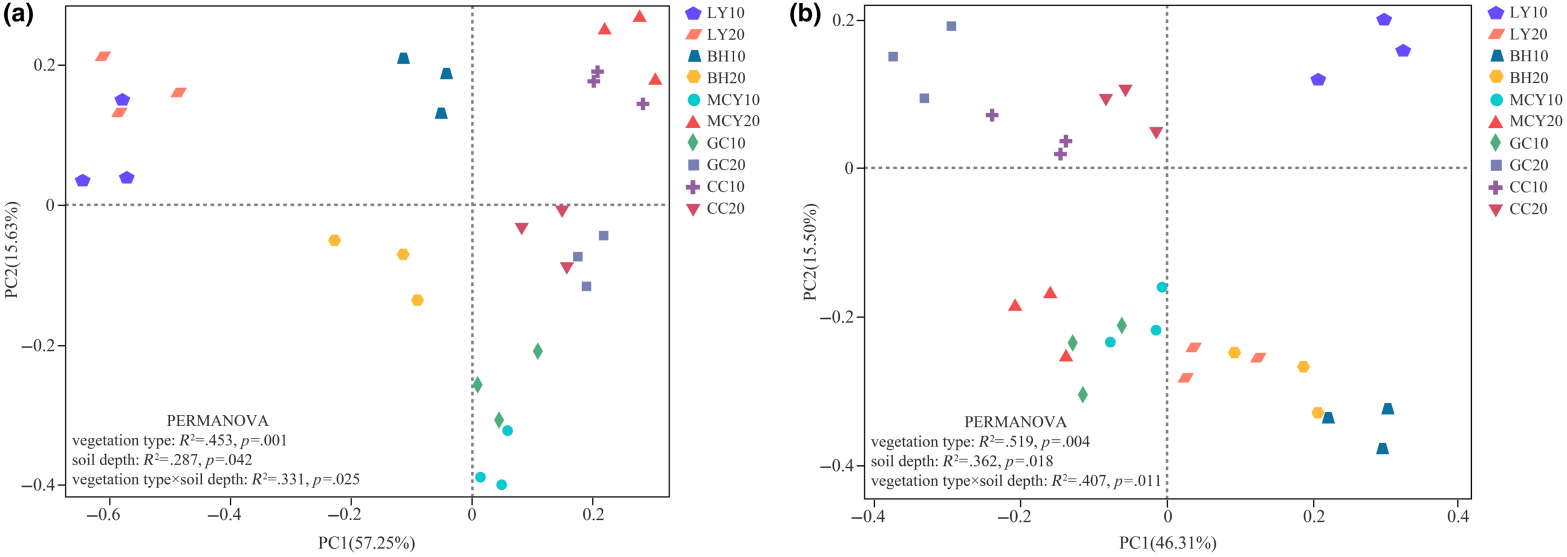
Principal coordinate analysis (PCoA) of soil microbial communities for soil samples in the five wetlands. (a) Bacterial communities; (b) fungal communities.

As shown in Figure 4a, the results showed PC1 and PC2 could explain 57.25 and 15.63% of the total variation in the bacterial communities, respectively. As for the fungal communities, the first two axes (PC1 and PC2 combined) explained 61.81% of the total variance (Figure 4b). Both the bacterial and fungal communities showed that the MCY, GC, and CC samples were grouped together, while the LY and BH samples were distinctly separated along the PC1 axis. PERMANOVA test revealed that vegetation type, soil depth, and their interactions exerted significant effects on bacterial and fungal community structure and that vegetation type explained more microbial community structure variation than soil depth.

### 3.3 Bacterial and fungal community composition

**FIGURE 5:**
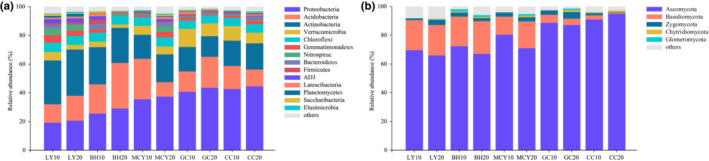
Microbial community composition at the phylum level of the different wetland soil samples. (a) Bacterial community relative abundance; (b) fungal community relative abundance.

Proteobacteria (19.3–44.6%), Acidobacteria (10.1–31.8%), Actinobacteria (14.3–32.2%), Verrucomicrobia (2.4–12.6%), and Chloroflexi (5.6–6.5%) were dominant bacterial phyla in the five wetlands (Figure 5a). The proportion of Proteobacteria was also lower in LY (28.2–43.2%) and BH (16.8–21.2%) than in the other wetland samples. Acidobacteria exhibited a significant higher relative abundance in BH and MCY, which was significantly higher at 0–10 cm than 10–20 cm depth in MCY, showing an opposite distribution trend in BH. Actinobacteria displayed a higher proportion in LY and BH, whereas the relative abundance of Verrucomicrobia was higher in GC and CC. There was no significant difference in the relative abundance of Chloroflexi among the different wetland soils. As for fungal communities at the phylum level, the relative abundance of Ascomycota accounted for more than 66% in all soil samples, which was absolutely dominating in the five wetlands (Figure 5b). Ascomycota (87.3–94.9%) showed a higher relative abundance in GC and CC, while a significantly lower proportion of Basidiomycota (0.7–5.6%) was observed in GC and CC than in the other wetland samples. Zygomycota (2.9–4.6%) was more abundant in GC, compared with the other samples.

**FIGURE 6:**
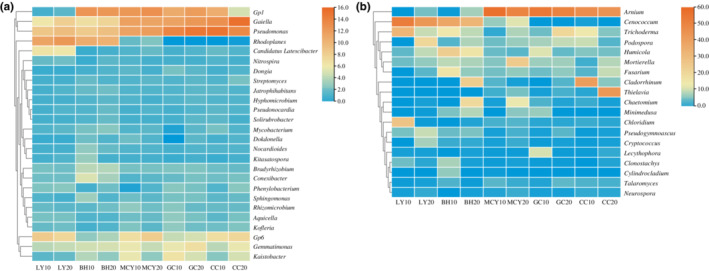
Heatmaps and hierarchical cluster diagrams of the relative abundances of dominant bacteria and fungi at the genus level of the different wetland soil samples. (a) Bacterial community; (b) fungal community.

Further comparison of the bacterial community at the genus level identified *Gaiella* (7.5–13.9%), *Pseudomonas* (7.7–13.0%), and *Gp1* (2.5–9.5%) as the most dominant genera in MCY, GC and CC (Figure 6a). *Rhodoplanes* (7.4–8.7%), *Pseudomonas* (4.9–5.5%), and *Gp6* (4.0–4.6%) were the dominant genera in LY. In BH, *Gp1* (8.7–9.1%), *Rhodoplanes* (6.2–6.8%), and *Pseudomonas* (4.6–5.3%) were the dominant genera. At the fungal genus level, *Arnium* displayed a higher proportion in MCY (38.7–54.5%), GC (40.1–46.3%), and CC (29.7–33.0%), but this genus represented a much lower fraction in LY (0.0–1.6%) and BH (0.1–2.9%) (Figure 6b). The proportion of *Cenococcum* was also higher in LY (28.2–43.2%) and BH (16.8–21.2%) than in the other wetland samples. *Thielavia* and *Cladorrhinum* showed high relative abundances in CC20 (28.2%) and CC10 (25.4%), but they were not detected in LY and BH, LY. Lastly, a significantly higher proportion of *Trichoderma* and *Chloridium* was observed in LY10 compared with the other samples.

### 3.4 Extracellular enzymatic activity

**FIGURE 7:**
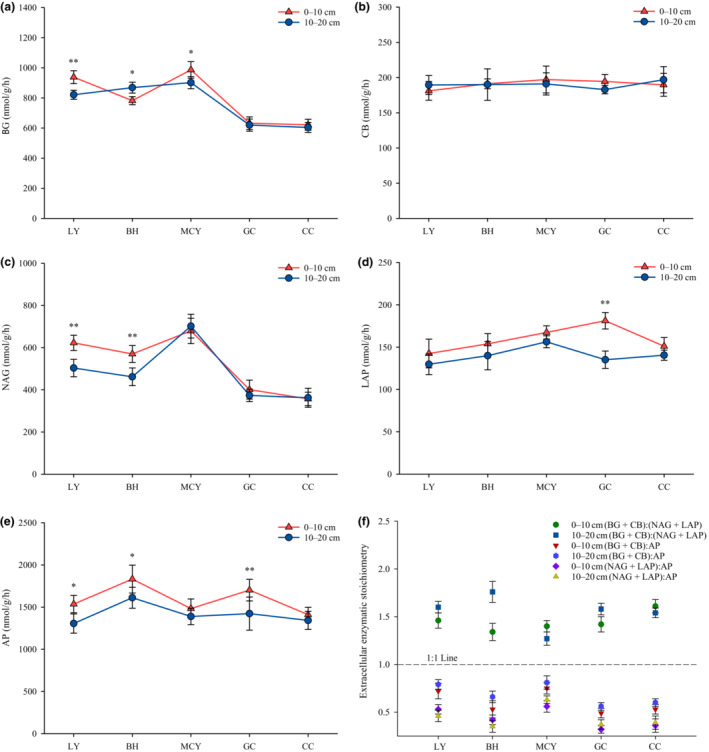
Extracellular enzymatic activities of soil samples from different wetlands and soil layers. (a) β‐glucosidase (BG); (b) cellobiohydrolase (CB); (c) β‐N‐acetylglucosaminidase (NAG); (d) leucine aminopeptidase (LAP); (e) acid phosphatase (AP); (f) extracellular enzymatic stoichiometry.

The activity of C‐cycling enzyme β‐glucosidase (BG) was significantly lower in GC and CC than in the other wetlands soils (Figure 7a), whereas there were no significant differences in activity of cellobiohydrolase (CB) among the samples (Figure 7b). The activity of N‐cycling enzyme β‐N‐acetylglucosaminidase (NAG) was highest in MCY, while it was significantly lower in both GC and CC, with similar and intermediate activity in LY and BH (Figure 7c). The activity of leucine aminopeptidase (LAP) was significantly higher in GC10 than the other soil samples (Figure 7d). The activity of P‐cycling enzyme acid phosphatase (AP) was significantly higher in BH, while in LY, BH and GC this enzyme was significantly lower at a depth of 10–20 cm compared with the 0–10 cm layer (Figure 7e). At both depths in the different wetland soils, the ratio of (BG + CB):(NAG + LAP), indicative of the C:N ratio, was above 1 and ranged from 1.27 to 1.76. Additionally, the C:P ratio, calculated as (BG + CB):AP, was below 1, with a range of 0.49–0.81. An even low range was found for N:P which was in the range of 0.32–0.63, as calculated by (NAG + LAP):AP (Figure 7f).

### 3.5 Relationships among soil properties, extracellular enzymatic activities, and microbial communities

**FIGURE 8:**
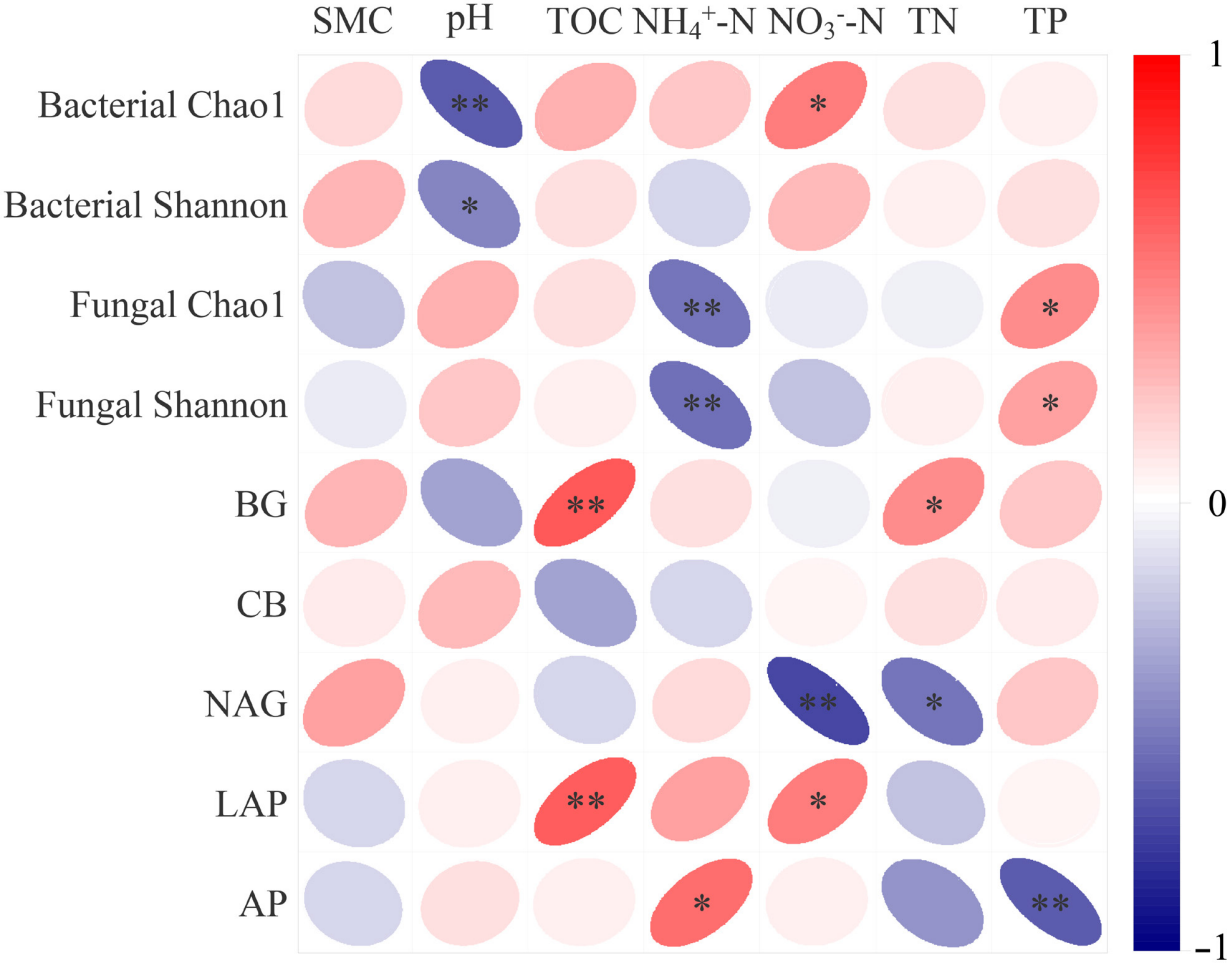
Pearson's correlation coefficients among soil physicochemical properties, microbial (bacterial and fungal) diversity, and extracellular enzymatic activities.

According to Pearson's correlation coefficients, pH was significantly negatively associated with bacterial Chao1 and Shannon indices, while ${\mathrm{NO}}_{3}^{-}$–N content was significantly positively related to bacterial Chao1 (*p* < .05) (Figure 8). Fungal Chao1 and Shannon indices were significantly positively associated with TP content, but significantly negatively related to ${\mathrm{NH}}_{4}^{+}$–N (*p* < .05). In addition, BG and LAP activities showed significant positive correlations with TOC, whereas NAG activity had a significant negative correlation with ${\mathrm{NO}}_{3}^{-}$–N (*p* < .01). AP activity was significantly negatively related to TP (*p* < .01), while there were no significant relationships between CB activity and soil variables.

**FIGURE 9:**
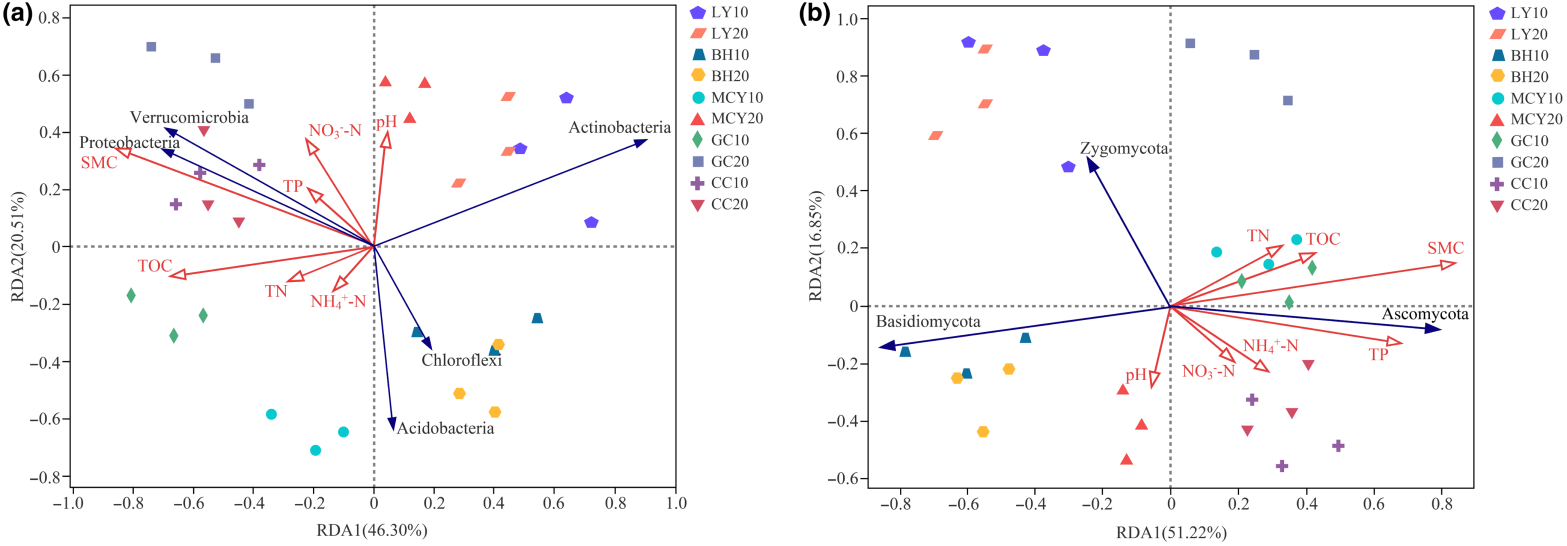
Redundancy analysis (RDA) of soil environmental factors and microbial community structure at the phylum level. (a) Bacterial community structure; (b) fungal community structure. The red and blue line with arrows indicates soil environmental factors and the dominant bacterial and fungal phyla, respectively.

Redundancy analysis (RDA) showed the first two axes accounted for 66.81% of the total variance in bacterial community structure (Figure 9a), whereas RDA1 and RDA2 explained 51.22 and 16.85% of the variation in fungal community structure (Figure 9b), respectively. Monte Carlo permutation tests demonstrated that SMC (*F* = 5.1, *p* = .006) and TOC (*F* = 3.9, *p* = .021) were the major contributors to the observed variations in bacterial community structure. Moreover, SMC (*F* = 6.8, *p* = .002) and TP (*F* = 4.7, *p* = .034) had significant influences on fungal community structure. Proteobacteria and Verrucomicrobia had positive correlations with SMC; Actinobacteria had a negative correlation with TOC; Acidobacteria was negatively associated with pH. Moreover, as for dominant fungi, Ascomycota was positively correlated with SMC and TP, while Basidiomycota was negatively correlated with SMC.

## 4 DISCUSSION

Our results revealed significant correlations between microbial alpha diversity (Chao1 and Shannon) indices and the determined soil parameters (pH, ${\mathrm{NO}}_{3}^{-}$–N, ${\mathrm{NH}}_{4}^{+}$–N, and TP), in agreement with other studies for different wetlands (Bickford et al., 2020; Briones et al., 2022; Kang et al., 2021). Soil pH is considered as a strong driver that can shape soil bacterial community structure in various ecosystems, which may influence the physiology, morphology, and metabolism of soil microorganisms (Shanmugam & Kingery, 2018). Similar to findings reported by Siles et al. (2016), we found that differences in fungal richness and diversity were explained by ${\mathrm{NH}}_{4}^{+}$–N and TP content, demonstrating that fungal diversity and abundance generally increased with higher phosphorus and lower nitrogen availability. Our data further indicated that microbial alpha diversity was not strongly associated with soil depth. This result may be partly attributed to that microbial groups showed high diversity and interconnectedness in high‐latitude permafrost regions, which was likely to make their communities show resistance or resilience to small‐scale (only for 0–10 cm and 10–20 cm soil layers) disturbances, contributing to maintain the stability of psychrophilic bacterial and fungal community diversity (Mondav et al., 2017).

The habitats varied from *Larix gmelinii* swamp to tussock swamp along the forest–wetland ecotone, and habitats with different vegetation characteristics have a regulatory effect on microbial community characteristics. A study by Pries et al. (2013) in Alaska permafrost regions indicated that vegetation type could significantly influence local soil environment through different litter inputs. The PCoA results suggested that bacterial and fungal communities in MCY, GC, and CC were significantly different from those in LY and BH. The needle litter of *Larix gmelinii* has a high lignin and cellulose content, as well as less simple and soluble substrates, while *Betula platyphylla* produces broadleaf litter that is more decomposable, which provides various nutrient resources for growth and reproduction of soil microorganisms (Prescott & Vesterdal, 2021). Due to the flat terrain with periodic ponding and a thin active layer, trees (mostly *Alnus sibirica* var. *hirsute*) were sparser in MCY, while *Sphagnum palustre* in thicket swamp and tussock swamp contain larger amounts of high molecular weight polyphenolic compounds, restricting microbial activity and presumably reshaping the microbial community (Brouns et al., 2016). Overall, these results confirmed our hypotheses, emphasizing the fundamental effects of vegetation variation on soil microbial community structure and diversity, which may be strongly correlated with soil physicochemical properties.

Most Proteobacteria typically found in soil are copiotrophs (*r*‐strategists) that are typically related to organic carbon‐rich substrates, whereas Actinobacteria and Acidobacteria members are more often oligotrophs (*k*‐strategists) that are capable of degrading a variety of recalcitrant forms of carbon (Ramin & Allison, 2019). Our observations are in accordance with the results of Heděnec et al. (2018) who also reported a higher relative abundance of Proteobacteria in soil with a higher soil moisture content, whereas Actinobacteria was more abundant at lower soil moisture contents. Acidobacteria showed a high relative abundance in BH20 and MCY10, presumably representing members of this phylum that are better adapted to lower pH environments (Pankratov et al., 2008). In comparison, the detected members of Chloroflexi were less sensitive to variation in wetland vegetation types and habitat conditions. We observed that the average relative abundance of Ascomycota for all soil samples exceeded 66.1%, suggesting that the Ascomycota by far dominate the fungal communities in these cold region wetlands. This is not surprising, since Ascomycota dominate soil fungal communities at the global scale (Egidi et al., 2019). Previous studies suggested that Ascomycota are better equipped to withstand environmental pressures, are more competitive than Basidiomycota, and can rapidly decompose and utilize complex organic compounds, thereby inhibiting the proliferation of Basidiomycota in anoxic environments. Such observations are also supported by our study (Lang et al., 2022; Park et al., 2020). Blanchette et al. (2010) indicated that cold‐tolerant *Thielavia* were pioneers in obtaining carbon from recalcitrant organic polymers in polar regions. Some fungi are more sensitive to vegetation type than bacteria, such as members of the genus *Cenococcum* were identified to form ectomycorrhizas with a variety of plants, and to mediate reciprocal carbon and nitrogen nutrient exchange between these hosts through mycorrhizal networks (Corrêa et al., 2011). As mentioned above, both bacterial and fungal communities respond differently along the assessed environmental gradients, as they have different survival strategies and cellular metabolism, and their differential responses would affect overarching resilience and resistance of the microbial communities in the forest–wetland ecotone.

The findings indicated that BG and NAG activities tended to be higher in LY, BH, and MCY than GC and CC, probably because tree‐dominated wetlands may provide more nitrogen‐rich woody litters and supply more root‐derived organic carbon to the microorganisms, thus enhancing this enzyme activity. Liu et al. (2021) and Menon et al. (2013) previously proposed that vegetation types determine the quality and quantity of litters on the top soil layers and affect the structure of microbial communities via the release of secretions and oxygen around plant roots, hence indirectly influencing extracellular enzymatic activities. We observed that the activity of AP was significantly higher in BH compared to the other wetlands and it was significantly lower at 10–20 cm depth than 0–10 cm in LY, BH and GC. Moreover, AP activity showed significant negative correlations with TP. Competition for available nutrients occurs between plants and microorganisms, and the combination of low phosphorus availability and high AP activity suggests that microorganisms increase the production of AP to meet their demand (Steinweg et al., 2018). In addition, the ratios of (BG + CB):AP and (NAG + LAP):AP are all below 1.0 in five wetland types, indicating a strong P limitation in our study area. Margalef et al. (2017) demonstrated that there is a large amount of organic matter in the boreal peatlands where P is trapped, so that plants and microorganisms are unable to utilize it.

Our study demonstrates that soil moisture content (SMC) is a key driver that explains the observed variations of soil bacterial and fungal communities, which is in accordance with studies in the Zoige wetland (Fan et al., 2022), alpine wetland (Li et al., 2022), and boreal fens (Peltoniemi et al., 2015). Soil moisture exerts effects on soil respiration, oxygen availability, soil nutrient diffusion, and litter decomposition, which further influences microbial community structure, particularly those related to carbon and nitrogen cycles in high‐latitude permafrost region wetland ecosystems (Ren et al., 2018). Extreme moisture conditions present stress for microbial communities, and various taxa exhibit a variety of physiological acclimation strategies such as production of microbial extracellular polymers, osmotic acclimation and reducing metabolism; as a consequence, microorganisms may consume more resources for survival rather than growth and multiplication (Cruz‐Paredes et al., 2021). Moreover, we also speculate that there may be the specific soil moisture threshold along the natural environmental gradients in the Nanweng River Nature Reserve. When wetland ecosystems exceed this threshold, both soil physicochemical parameters and microbial communities quickly reach a new stable state, as likewise observed by Frindte et al. (2019) in a study of Arctic‐alpine regions. In addition to soil moisture, the soil bacterial and fungal community composition was also affected by TOC and TP, respectively. Previous studies have proved that at the Arctic landscape scale, TOC was recognized as the major factor affecting bacterial community structure and was closely related to soil moisture (Malard et al., 2021). Furthermore, fungi can degrade complex molecules present in plant litter, comprising lignin and cellulose components, but they require soil phosphorus for this, which might corroborate the crucial role of soil TP content in influencing fungal community structure. Future climate change will further induce such alterations in soil abiotic conditions and can result in rapid shifts in plant communities of high‐latitude permafrost region wetlands, which has cascading effects on underground decomposers and carbon pool turnover. Because vegetation type plays pivotal roles in affecting soil microbial communities, future research has to take into account plant‐derived factors such as plant diversity, biomass, root structure, and functional types, illuminating the main causal relationships among wetland plant communities, soil microorganisms, and carbon fluxes in fragile permafrost ecosystems.

## 5 CONCLUSION

Our results provided empirical evidence that vegetation type and soil depth shape the soil microbial community structure, and we individuated major soil environmental factors explaining these changes. Soil microbial community structure was largely influenced by vegetation type and was almost unaffected by soil depth. Additionally, our study suggested that SMC and TOC were the most important variables changing the soil bacterial community structure, while SMC and TP were the key drivers of altered soil fungal community structure. Changes in soil extracellular enzymatic activities were more closely associated with soil properties (TOC, ${\mathrm{NO}}_{3}^{-}$–N, and TP) than with microbial community composition. For future work, it is necessary to investigate the resilience and resistance of cold region wetland ecosystem functions to high‐latitude permafrost degradation, as well as to describe the impact mechanisms of vegetation species composition on belowground microbial and enzymatic processes.

## AUTHOR CONTRIBUTIONS

**Lingyu Fu:** Conceptualization (equal); data curation (equal); investigation (equal); writing – original draft (equal); writing – review and editing (equal). **Ruifeng Xie:** Conceptualization (equal); data curation (equal); software (equal); writing – review and editing (equal). **Dalong Ma:** Conceptualization (equal); data curation (equal); methodology (equal); writing – review and editing (equal). **Man Zhang:** Data curation (equal); investigation (equal); methodology (equal). **Lin Liu:** Data curation (equal); software (equal); writing – original draft (equal).

## CONFLICT OF INTEREST STATEMENT

The authors declare that they have no conflict of interest.

## Data Availability

Raw sequences were deposited in the National Center for Biotechnology Information (NCBI) public database under the accession numbers of PRJNA918310 for bacteria and PRJNA918495 for fungi.
